# Prevalence of HPV-DNA and E6 mRNA in lung cancer of HIV-infected patients

**DOI:** 10.1038/s41598-022-17237-5

**Published:** 2022-08-01

**Authors:** Guillem Sirera, Sebastián Videla, Verónica Saludes, Eva Castellà, Carolina Sanz, Aurelio Ariza, Bonaventura Clotet, Elisa Martró

**Affiliations:** 1 Fight AIDS Foundation, Germans Trias i Pujol University Hospital, Badalona, Spain; 2 Infectious Diseases Department, Germans Trias i Pujol University Hospital, Badalona, Spain; 3 Clinical Research Support Unit (HUB-IDIBELL: Bellvitge University Hospital & Bellvitge Biomedical Research Institute), Clinical Pharmacology Department, Bellvitge University Hospital, L’Hospitalet de Llobregat, Spain; 4 Pharmacology Unit, Department of Pathology and Experimental Therapeutics, School of Medicine and Health Sciences, IDIBELL, University of Barcelona, Barcelona, Spain; 5 Microbiology Department, Laboratori Clinic Metropolitana Nord, Germans Trias i Pujol University Hospital, Germans Trias i Pujol Research Institute (IGTP), Badalona, Spain; 6 Consorcio de Investigación Biomédica en Red de Epidemiología y Salud Pública (CIBERESP), Instituto de Salud Carlos III, Madrid, Spain; 7 Department of Pathology, Germans Trias i Pujol University Hospital, Badalona, Spain; 8 IrsiCaixa AIDS Research Institute, University Hospital Germans Trias i Pujol, Badalona, Spain; 9 Universitat de Vic–Universitat Central de Catalunya (UVIC-UCC), Vic, Spain; 10 Department of Medicine, Universitat Autònoma de Barcelona, Bellaterra, Spain

**Keywords:** Human papilloma virus, Viral infection

## Abstract

HIV-infected individuals could be at a greater risk for developing lung cancer than the general population due to the higher prevalence in the former of human papillomavirus (HPV) in the oral cavity and higher smoking rates. Our aim was to assess HPV prevalence and E6 viral oncogene transcription in lung cancer samples from HIV-infected individuals. This was a single-center, retrospective study of a cohort of HIV-1-infected patients diagnosed with and treated for lung cancer. Pathological lung samples archived as smears or formalin-fixed paraffin-embedded blocks were subjected to HPV genotyping, detection of human p16 protein and assessment for HPV E6 mRNA expression*.* Lung cancer samples from 41 patients were studied, including squamous cell carcinoma (32%), adenocarcinoma (34%), non-small cell cancer (27%), and small cell cancer (7%). HPV DNA was detected in 23 out of 41 (56%, 95% CI 41–70%) of samples and high-risk (HR)-HPV types were detected in 16 out of 41 (39%, 95% CI 26–54%), HPV-16 being the most prevalent [13/16 (81.3%, 95% CI 57.0–93%]. In samples with sufficient material left: expression of p16 was detected in 3 out of 10 (30%) of HR-HPV DNA-positive tumors and in 3 out of 7 (43%) of the negative ones; and E6 mRNA was detected in 2 out of 10 (20%) of HPV-16-positive samples (squamous lung cancers). These two patients had a background of a previous HPV-related neoplasia and smoking. HR-HPV DNA detection was prevalent in lung cancers in HIV-infected patients. However, viral oncogene expression was limited to patients with previous HPV-related cancers.

## Introduction

A significant decrease in morbidity and mortality associated with HIV infection, as well as in the incidence of AIDS-defining cancers, has been achieved since the introduction of antiretroviral therapy (ART)^1^. However, there has been a parallel increase in the incidence of non-AIDS cancers^1–7^, which constitute a heterogeneous group of neoplasms including, among others, cancers of the lung, liver, anus, head, neck, skin, and Hodgkin’s lymphoma. A low CD4 count at diagnosis, the duration of previous ART treatment, smoking, and age at diagnosis have been described as predictors of the appearance of non-AIDS cancers^5^. Recent estimations suggest that by 2030 lung, prostate, liver, and anus cancers will be the most frequent cancers in the USA in HIV-infected individuals^8^. Because these cancers may impact life expectancy in people living with HIV^9^, the prevention of cancer among HIV-positive individuals is essential.

Among the population of people living with HIV, the risk of lung cancer has been estimated to be two to sevenfold higher and its progression faster than in the general population. Though younger average age and a higher smoking rate may contribute to this increased risk^10–15^, other factors are directly associated with HIV-infection, such as diminished immune surveillance, the activation of proto-oncogenes, or the inhibition of tumor suppressor genes that would lead to oncogenesis^16,17^. On the other hand, views differ sharply regarding the relationship between the CD4 cell number or duration of immunodeficiency and the risk of lung cancer^18,19^. Chronic pulmonary inflammation has been associated with an increased risk of lung cancer^20–23^, and a higher risk of chronic obstructive pulmonary disease has been observed among the HIV-positive population due to its high smoking rate. Therefore, HIV infection may also predispose to the development of cancer^24^.

The carcinogenicity of human papillomavirus (HPV) has been well established. High-risk (HR) HPV types are responsible for virtually all cervical cancer cases and are also associated with a fraction of cancers of the vulva, vagina, penis, anus, as well as head and neck cancers^25^. Several meta-analyses have explored the association between HPV and lung cancer in the non-HIV-infected population, but it remains controversial^26–28^, and the few studies that have assessed the expression of HPV oncogenes in lung cancer are inconclusive or have not found E6/E7 mRNAs^29–32^.

The prevalence of HPV in the oral cavity has been estimated to be near 7% for the general population^33^ and 14% for the HIV-infected population^34^. The HPV-16 genotype is the most prevalent among HIV-infected individuals^34–36^. Practicing oral sex has been shown to increase four to sixfold the risk of HPV infection^37^, possibly contributing to the spread of the virus through the air to the lungs. Among the population of people living with HIV, the higher HPV prevalence in the oral cavity together with their high smoking rates could place them at greater risk for developing lung cancer. Our aim was to assess HPV prevalence and E6 viral oncogene transcription in lung cancer samples from HIV-infected individuals. In order to do this, lung cancer samples were tested for the presence of HPV DNA and the expression of the viral oncogene E6.

## Patients and methods

### Study design

The study was a single-center, retrospective cohort study based on HIV-1-infected patients with lung cancer which had been diagnosed and treated in our tertiary hospital. This center is the referral hospital for 1,200,000 local residents in its catchment area and general hospital for 200,000 inhabitants of the Northern Barcelona metropolitan area.

The study was performed in accordance with the stipulations of the Declaration of Helsinki. The protocol was approved by the local IRB (Comitè d’Ètica de la Investigació Clínica at Germans Trias i Pujol University Hospital) and a waiver for written informed consent was given. After IRB approval, prospective analysis of the lung biopsy samples for HPV detection and typing with the aim of studying HPV’s role in lung cancer was performed. An appropriate level of confidentiality, in terms of the protection of personal data as required by Spanish Law (LOPD 3/2018), was also ensured.

### Study population and clinical specimens

The patients included in this cohort study had to fulfill the following criteria: age ≥ 18 years, either sex, positive HIV serology results before lung cancer diagnosis, and availability of the specimens used for the diagnosis. The following variables were collected by medical records review: date of birth, gender, lifetime use of injected drugs, sexual orientation, smoking status, age at HIV infection diagnosis, age at lung cancer diagnosis, histological type of lung neoplasm (by histology or cytology), number of CD4 nadir (the lowest point previous to lung cancer diagnosis) and CD4 lymphocytes (at the moment of lung cancer diagnosis), HIV viral load at the time of lung cancer diagnosis, type of ART, AIDS prior to lung cancer (yes/no), evolution (alive, lung cancer relapse, died/cause of death) up to May 31st, 2020, and diagnosis of other neoplasms.

The pathological specimens were obtained by bronchoscopy (bronchial aspirate, bronchoalveolar lavage, or biopsy) or by endobronchial ultrasound bronchoscopy (EBUS) fine-needle aspiration cytology (FNAC) of the mediastinal metastatic lymph nodes. Biopsies were archived in Formalin-Fixed Paraffin-Embedded (FFPE) blocks. The aspirates (BAS, BAL, lung or EBUS-FNAC) were recovered and smeared on slides, fixed with 95% ethanol and stained with Papanicolaou stain. Additional material was obtained and processed as cell blocks for ancillary studies. Cell blocks were prepared by air-drying the slides to clot and scraping them into 10% formalin for subsequent processing in the laboratory. Cell blocks were then embedded in paraffin.

### HPV identification

In order to assess HPV presence and transcriptional activity in lung cancer, the following tests were performed: (i) all lung cancer specimens were subjected to HPV DNA testing and genotyping; (ii) in HPV-positive specimens with sufficient sample left, new sample sections and nucleic acid extractions were performed to assess the expression of E6 mRNA; and (iii) human p16 protein (also known as cyclin-dependent kinase inhibitor 2A) expression was assessed by immunohistochemistry in those HPV-positive samples with sufficient material left. Additionally, p16 was assessed in HPV-negative samples with sufficient sample left in order to check for specificity and, if positive, E6 mRNA was also tested for.

#### DNA extraction from clinical specimens

FFPE samples, including biopsies and some cytologies, were checked for representativity and selection of the tumor area by hematoxylin–eosin staining. FFPE blocks were cut into 5 μm-thick sections under strict conditions to avoid cross-contamination. Blocks containing non-HPV related cancers were also included and used as negative controls. Sections were deparaffinized with xylol and washed with ethanol. When possible, tissue was manually macro-dissected for tumor cell enrichment. For cytology samples not included in paraffin, the DNA extraction was performed using Papanicolaou-stained smears, from which cells were recovered. DNA was then isolated using QIAamp DNA microkit (Qiagen GmbH, Hilden, Germany) following manufacturer’s instructions. For FFPE samples, an initial step of 90 °C for 1 h after overnight proteinase K digestion was included. The integrity of the extracted DNA was checked by PCR using the multiplex “BIOMED-2 Control Gene” set of primers (PCR products of 100, 200, 300, and 400 pb) followed by agarose gel electrophoresis.

#### HPV DNA detection and genotyping

Extracted DNA was subjected to HPV detection and genotyping using the following molecular methods:

(i) *Anyplex™ II HPV28 real-time PCR* All samples were first tested with the Anyplex™ II HPV28 assay (Seegene, Seoul, South Korea), which relies on the newly developed TOCE™ (tagging oligonucleotide cleavage and extension) and DPO™ (dual priming oligonucleotide) technologies to detect and genotype in a single step 28 different types of HPV in two real-time PCR multiplex reactions per specimen followed by melting curve analysis. The targeted HPV DNA region and length are proprietary information of Seegene. This assay is able to detect HPV types 6, 11, 16, 18, 26, 31, 33, 35, 39, 40, 42, 43, 44, 45, 51, 52, 53, 54, 56, 58, 59, 61, 66, 68, 69, 70, 73, and 82. In parallel, an endogenous internal control (human β-globin gene) is amplified in each PCR reaction to ensure sample quality and to exclude PCR inhibition. Reactions were performed according to manufacturer’s instructions in a CFX96 real-time thermocycler (Bio-Rad, Hercules, CA, USA). This assay has been previously validated to detect and genotype HPV in FFPE biopsies from genital lesions from patients with a clinical suspicion of HPV infection^38^ and FFPE biopsies from oropharyngeal carcinomas^39^.

(ii) *INNO-LiPA HPV genotyping* All samples were then tested with this assay, which uses consensus primers to amplify a 65 bp region in the HPV L1 gene by conventional PCR. Genotyping was then performed by automated reverse hybridization with the INNO-LiPA HPV Genotyping Extra assay (Fujirebio, Ghent, Belgium), according to manufacturer’s instructions. This assay is able to detect the following HPV types: HPV6, 11, 16, 18, 26, 31, 33, 35, 39, 40, 43, 44, 45, 51, 52, 53, 54, 56, 58, 59, 66, 68, 69, 70, 71, 73, 74 and 82 (it is not able to discriminate between HPV69 and 71). Specimens that were positive for HPV DNA but were non-typeable with this assay were coded as HPV-X (unknown genotype) and were further tested with the INNO-LiPA HPV Genotyping Extra II (Fujierbio, Ghent, Belgium). The INNO-LiPA also amplifies a 280 bp fragment of the human HLA-DPB1 gene to verify DNA quality and extraction efficiency and uses uracil-N-glycosylase to avoid contamination by PCR products.

(iii) *In-house HPV16-specific and generic PCRs* Specimens with discordant results for HPV16 by the HPV28 assay and any of the two INNO-LiPA assays were subjected to an in-house type-specific PCR assay targeting the E6 region^40^. In addition, in order to genotype specimens with an HPVX result by the INNO-LiPA assays, and to assess HPV DNA presence in specimens that were negative by commercial assays, the GP5 + /GP6 + consensus primers for alpha papillomaviruses targeting the L1 gene was used^41^. PCR reactions were performed in 50 µL containing 0.4 mM of dNTPs, 1 µM of each primer, 3 mM MgCl_2_, 5 × Go Taq Flexi buffer and 1 U of GoTaq Flexi DNA Polymerase (Promega). As described previously^41^ thermocycler conditions were: 2 min at 95 °C; 40 cycles of 94 °C for 20 s, 53 °C for 20 s, and 72 °C for 40 s; then 72 °C for 7 min. PCR products were visualized by capillary electrophoresis (Qiaxcel, Qiagen) checking for bands of 150 bp (generic HPV PCR), and 120 bp (HPV-16 PCR). Generic PCR amplified products were then directly sequenced with the GP5 + /GP6 + primers. Readings were assembled and edited with the STADEN package v2.0. Sequences obtained were compared to available HPV sequences in the National Center for Biotechnology Information (NCBI) database by using the BLAST algorithm (http://www.ncbi.nih.gov/BLAST). Two FFPE biopsies from two HPV16-positive cervical cancer cases were used as positive controls. A negative control was also included in each run.

### Expression of HPV E6 mRNA

In order to assess the active transcription of HPV E6 oncogene where either HPV DNA or human p16 was detected, new sections were obtained, and hematoxylin–eosin staining was performed in the last section to confirm the presence of the tumor (Table 3). Then, the presence of E6 mRNA was assessed. Briefly, RNA was extracted from tissue sections and subjected to a previously developed assay (HPV E6*I/Ubc RT-PCR), in order to amplify the E6*I transcript of HPV16 or other non-HPV16-types by hybridization to probes that are coupled to Luminex beads^42^. This splice-site specific assay is able to detect transcriptional activity of 12 high-risk (HR)-HPV (IARC Group 1) and eight probable/possible high-risk (pHR)-HPV types (IARC Group 2A/B carcinogens). Cellular ubiquitin C (UbC) transcripts we also amplified as an RNA quality control.

### Expression of human p16 protein

This technique was performed on HPV DNA positive specimens with sufficient sample left after mRNA testing, and also on HPV-negative samples with sufficient sample left in order to check for specificity. Immunohistochemistry was performed for p16 using the E6H4 antibody (Ventana Medical Systems, Inc.) with antigen retrieval consisting of 50 min at pH 6. The dilution of the antibody was 1:10. Both nuclear and cytoplasmic staining was required for a cell to be considered “positive” and staining distribution was read as negative when no staining or staining in less than 70% of tumor cells and positive when staining in > 70% of tumor cells regardless of intensity^43^.

### Statistical analysis

#### Sample size

Due to the exploratory nature of our study, no formal calculation of sample size was performed. The final sample size was defined as the number of HIV-1-infected patients who fulfilled the inclusion criteria.

#### Statistical procedures

Baseline characteristics were summarized using standard descriptive statistics, and a descriptive analysis was carried out.

The overall prevalence of HPV infection and the prevalence of HR-HPV types (i.e. 16, 18, 31, 33, 35, 39, 45, 51, 52, 56, 58, 59), which have been classified as class 1 carcinogens in humans^44^, were estimated and 95% confidence intervals (95%CI) were calculated. Prevalence was defined as detection by at least one experimental method, based on the fact that assays for the detection of HPV DNA from FFPE specimens may differ in sensitivity due to (i) the length of the PCR amplicon (DNA may be split into < 200 bp fragments in archival samples), (ii) the region targeted in the HPV genome (L1 may be lost upon integration), (iii) HPV types included in the assay design, and (iii) specific conditions of each assay. Cross-contamination events between samples was ruled out by including negative controls in each run. Data analysis was carried out using SPSS version 20.

## Results

### Patient characteristics

Between January 1983 and December 2014, 51 HIV-1-infected patients were diagnosed with lung cancer, of whom 41 had an archival tumor sample available in the Pathology Department of our hospital. Therefore, HPV analysis in lung cancer was performed on 41 lung samples from these 41 patients. Table 1 shows patient characteristics. No significant differences were found between the general characteristics of these 41 patients and those for which HPV testing was not performed (data not shown). The diagnosis of lung neoplasm was performed from a biopsy in 26 (63%) of patients, and from cytology in the remaining 15 (37%). The histological/cytological types of lung neoplasm were squamous in 13 (32%) patients, adenocarcinoma in 14 (34%), non-small cell in 11 (27%), and small cell in 3 (7%). Prior to the diagnosis of lung cancer (baseline characteristics), only 2 out of 41 patients had a background of another cancer (squamous neoplasia of vulva and squamous neoplasia of anal canal).

**Table 1:** Demographic and clinical characteristics of HIV-1-infected patients with lung cancer.

HIV-1-infected patients with lung cancer (N = 41)	
**Gender**	
Men/women [n (%)]	37 (90)/4 (10)
**Age at HIV diagnosis, years**	
Overall mean (range)	36 (20–55)
Men/women mean (range)	37 (25–35)/29 (20–35)
**Age at diagnosis of lung cancer, years**	
Overall mean (range)	48.3 (36–64)
Men/women mean (range)	47 (37–64)/48 (44–54)
**Time between HIV and lung cancer diagnosis**	
Overall mean (range)	10 (1–22)
Men/women mean (range)	10 (1–18)/19 (13–24)
**AIDS (yes)**	
[n (%)]	16 (39)
**Patients with history of intravenous drug use**	
[n (%)]	23 (56)
**Sexual orientation of male patients**^**1**^** [n (%)]**	
MSM	8 (21.6)
Heterosexual	26 (70.2)
Not recorded	3 (8.2)
**Smoking status [n (%)]**	
Non-Smoker	0 (0)
Smoker up to lung cancer diagnosis	36 (87.8)
Ex-smoker	5 (12.2)
**CD4 counts (cells/mL)**	
At lung cancer diagnosis [mean (range)]	308 (5–1305)
Nadir [mean (range)]	134 (5–390)
Nadir lower than 200 [n (%)]	29 (70)
**On ART at lung cancer diagnosis**	
[n (%)]	25 (61)
**HIV viral load at lung cancer diagnosis**	
Lower than 40 copies/mL [n (%)]^2^	23 (56.1)

*MSM* men who have sex with men, *ART* antiretroviral therapy.

^1^The four women were heterosexual.

^2^Two patients on ART with detectable HIV viral load had a viral load of 27,168 and 540,000 copies/mL.

As of May 31st, 2020, none of the study participants had been diagnosed with other neoplasms, 35 (85.4%) had had a lung cancer-related death, three were alive and three had been lost to follow-up.

### HPV DNA detection and genotyping

The results obtained by the different HPV detection/genotyping techniques used, including the pooled results (positive by any of the assays used) and consistency of findings, are shown in Table 2. The internal control was detected in all samples (except for one that was inhibited) analyzed by the Anyplex™ II HPV28, and the HLA-DPB1 control gene was detected in all samples tested by the INNO-LiPA assay (including the one inhibited by the previous assay, which was positive). Overall, HPV DNA was detected in 23 out of 41 (56.1%, 95% CI 41–70%) of lung cancers; specifically, in 12 out of 26 (46.2%) of FFPE biopsies and 11 out of 15 (73.3%) of cytologies. In 21 out of 23 (91.3%) of those lung cancers positive for HPV DNA, it was detected by at least two assays. While the Anyplex™ II HPV28 assay detected 5 out of 23 (21.7%) of HPV DNA positive samples, the INNO-LiPA assay detected 21 out of 23 (91.3%) of them; however, in most of the cytology samples the latter assay was unable to genotype the HPV DNA detected (recorded as HPV-X).

**Table 2:** HPV results according to the different detection methods used.

ID	Sample type	Histological type	HPV28 (Seegene)	INNO-LiPA (Fujirebio)^a^	INNO-LiPAv.II (Fujirebio)^b^	HPV16-specific PCR^c^	α-HPV PCR and sequencing^d^	Pooled results	Consistency of HPV DNA findings^e^
1	Biopsy	Squamous	***16***	***16***	–	–	–	***16***	**2/2**
11	Cytology^f^	Squamous	***16***	***16***	–	–	–	***16***	**2/2**
18	Cytology^f^	Adenocarcinoma	ND	***16***	–	***16***	–	***16***	**2/3**
19	Biopsy	Non-small cell	ND	***16***	–	***16***	–	***16***	**2/3**
2	Biopsy	Squamous	***16***	***16***	–	–	–	***16***	**2/2**
24	Biopsy	Adenocarcinoma	ND	ND	–	–	ND	ND	
27	Biopsy	Adenocarcinoma	ND	ND	–	–	ND	ND	
28	Biopsy	Adenocarcinoma	ND	ND	–	–	ND	ND	
35	Biopsy	Non-small cell	ND	ND	–	–	**HPVX**	**HPVX**	**1/3**
4	Biopsy	Squamous	ND	***16***	–	***16***	–	***16***	**2/3**
6	Biopsy	Adenocarcinoma	ND	***16***	–	***16***	–	***16***	**2/3**
7	Biopsy	Squamous	ND	***16***	–	***16***	–	***16***	**2/3**
8	Biopsy	Adenocarcinoma	ND	***16***	–	***16***	–	***16***	**2/3**
9	Biopsy	Squamous	***33***	***16, 33, (52), (54)***	***16, 33***			***16, 33***	**16: 2/3, 33: 3/3**
3	Biopsy	Small cell	***16***	***16***	–	–	–	***16***	**2/2**
5	Biopsy	Squamous	ND	***16***	–	***16***	–	***16***	**2/3**
10	Cytology^f^	Adenocarcinoma	ND	**HPVX**	***18***	–	–	***18***	**1(2)/3**
17	Cytology^f^	Non-small cell	Inhibited	**HPVX**	**HPVX**	–	***58***	***58***	**1(3)/4**
12	Cytology	Small cell	ND	**HPVX**	**HPVX**	–	**HPVX**	**HPVX**	**3/4**
13	Cytology	Non-small cell	ND	**HPVX**	**HPVX**	–	ND	**HPVX**	**2/4**
14	Cytology	Squamous	ND	**HPVX**	**HPVX**	–	***56***	***56***	**1(3)/4**
15	Cytology	Adenocarcinoma	ND	**HPVX**	**HPVX**	–	**HPVX**	**HPVX**	**3/4**
16	Cytology	Non-small cell	ND	**HPVX**	**HPVX**	–	ND	**HPVX**	**2/4**
20	Cytology	Adenocarcinoma	ND	**HPVX**	**HPVX**	–	***16***	***16***	**1(3)/4**
21	Cytology	Adenocarcinoma	ND	**HPVX**	**HPVX**	–	**HPVX**	**HPVX**	**3/4**
22	Biopsy	Adenocarcinoma	ND	ND	–	–	ND	ND	
23	Biopsy	Non-small cell	ND	ND	–	–	ND	ND	
25	Biopsy	Squamous	ND	ND	–	–	ND	ND	
26	Biopsy	Non-small cell	ND	ND	–	–	**10**	**10**	**1/3**
29	Biopsy	Adenocarcinoma	ND	ND	–	–	ND	ND	
30	Biopsy	Squamous	ND	ND	–	–	ND	ND	
31	Biopsy	Squamous	ND	ND	–	–	ND	ND	
32	Biopsy	Adenocarcinoma	ND	ND	–	–	ND	ND	
33	Cytology^f^	Squamous	ND	ND	–	–	ND	ND	
34	Biopsy	Non-small cell	ND	ND	–	–	ND	ND	
36	Biopsy	Squamous	ND	ND	–	–	ND	ND	
37	Biopsy	Small cell	ND	ND	–	–	ND	ND	
38	Biopsy	Non-small cell	ND	ND	–	–	ND	ND	
39	Cytology	Non-small cell	ND	ND	–	–	ND	ND	
40	Cytology	Non-small cell	ND	ND	–	–	ND	ND	
41	Cytology	Adenocarcinoma	ND	ND	–	–	ND	ND	

*ND* not detected, – not performed, High-risk HPV types are marked in bold italics^47^.

^a^Due to INNO-LiPA band patterns obtained, the presence of HPV types in parentheses could not be confirmed.

^b^This assay was used to resolve non-typeable INNO-LiPA results.

^c^This assay was used to test specimens for which the HPV28 and INNO-LiPA assays yielded discordant results for HPV16.

^d^This strategy was used to genotype specimens whenever the INNO-LiPA assays yielded an HPVX, or to assess HPV DNA presence in specimens that were identified as negative by commercial assays.

^e^Number of assays with a positive result for a given HPV type among all assays performed. When a specific genotype was called by one of the assays, but a non-typeable result (HPVX) was obtained by the other assays, the numbers of positive but non-typeable results are given in parentheses.

^f^Cytology paraffin blocks.

As the HPV-X samples could not be classified into either low-risk or HR-HPV types, we assessed the prevalence of confirmed HR-HPV types among all the samples, which was 16 out of 41 (39.0%, 95% CI 25.7–54.3%), including 10 out of 26 (38.5%) of FFPE biopsies and 6 out of 15 (40.0%) of cytologies. The Anyplex assay detected 5 out of 12 (41.7%) of the HR-HPV types detected by the INNO-LiPA assay. Among all HR-HPV types, HPV-16 was the most frequently detected (13 out of 16, 81.3%). A mixed infection with two different genotypes (HPV-16 and -33) was detected in one case, and the other high-risk types detected were HPV-18, -56, and -58.

In terms of the histological/cytological types of lung neoplasm, HPV DNA was detected in 8 out of 13 (61.5%) of squamous carcinomas, in 7 out of 14 (50%) of adenocarcinomas, in 6 out of 11 (54.5%) of non-small cell cancers, and in 2 out of 3 (66.7%) of small cell lung cancers. The presence of HR-HPV types was confirmed in 8 out of 13 (61.5%) of squamous, 5/14 (28.6%) of adenocarcinoma, 1 out of 3 (33.3%) of small cell, and 2 out of 11 (18.2%) of non-small cell histological types.

### Detection of human p16 protein and expression of HPV E6 mRNA in selected samples

As HPV mRNA testing was prioritized over human p16 testing on HPV-positive specimens, the latter method could only be performed in 10 samples with enough material left (Table 3). Among them, 3 out of 10 (30%) were p16 positive (2 squamous and 1 adenocarcinoma). Additionally, p16 immunohistochemistry was performed on HPV DNA negative specimens with enough material left, and 3 out of 7 (43%) were positive (all of them adenocarcinomas).

**Table 3:** Detection of human p16, HPV DNA, and HPV E6 mRNA in selected specimens.

ID	Sample type	Histological type	Pooled HPV DNA results^a^	HPV E6 mRNA	p16 IHC
1	Biopsy	Squamous	**16**	**16**	Positive
2	Biopsy	Squamous	**16**	**16**	Positive
4	Biopsy	Squamous	**16**	ND	ND
5	Biopsy	Squamous	**16**	Invalid	ND
11	Cytology^b^	Squamous	**16**	ND	–
18	Cytology^b^	Adenocarcinoma	**16**	ND	ND
19	Biopsy	Non-small cell	**16**	ND	–
6	Biopsy	Adenocarcinoma	**16**	ND	Positive
7	Biopsy	Squamous	**16**	ND	ND
8	Biopsy	Adenocarcinoma	**16**	ND	ND
3	Biopsy	Small cell	**16**	Invalid	–
9	Biopsy	Squamous	**16, 33**	ND	ND
10	Cytology^b^	Adenocarcinoma	**18**	ND	–
17	Cytology^b^	Non-small cell	**58**	ND	ND
35	Biopsy	Non-small cell	**HPVX**	ND	–
24	Biopsy	Adenocarcinoma	ND	ND	Positive
27	Biopsy	Adenocarcinoma	ND	ND	Positive
28	Biopsy	Adenocarcinoma	ND	ND	Positive
23	Biopsy	Non-small cell	ND	–	ND
25	Biopsy	Squamous	ND	–	ND
29	Biopsy	Adenocarcinoma	ND	–	ND
30	Biopsy	Squamous	ND	–	ND

*IHC* immunohistochemistry, *ND* not detected, – not performed.

^a^Pooled HPV DNA results from Table 2.

^b^Cytology paraffin blocks.

In those samples where either HPV DNA or human p16 were positive and there was enough material left (14 biopsies and 4 cytologies), new sections were obtained. Among them, only two specimens (squamous lung cancers) were positive for E6 mRNA (Table 3), representing 2 out of 10 (20.0%) of HPV-16 positive samples tested for mRNA for which valid results were obtained (invalid results were obtained in another two cases). These two individuals had had a neoplasia due to HPV in other locations and had a background of smoking: a woman with squamous vulvar neoplasia and inguinal lymph node metastasis, who had undergone surgery three years earlier, and a man with an anal canal neoplasia diagnosed two years previously. In both patients, the primary HPV-related cancer was HPV-16-positive, detected at the time of diagnosis (data extracted from medical records). Specimens of the previous cancer and metastasis were available only in the case of the woman. HPV-16 DNA was detected again in the biopsies of the vulvar neoplasm and the inguinal metastasis. Likewise, E6 mRNA was also detected in these two biopsies.

## Discussion

The causal relationship between HPV infection and lung cancer is currently controversial. To the best of our knowledge, this is the first study to assess the presence of HPV DNA and viral expression markers in lung cancer tissue from HIV-infected individuals.

HPV DNA and HR-HPV DNA were detected in 56% and 39%, respectively, of the lung cancers of HIV-positive individuals examined in this study. This proportion is in the upper range of the proportions reported in previous meta-analyses including HIV-negative individuals with lung cancer from European countries (0% to 40.0%)^27,28^. HPV-16 was the most frequently detected type (57% among all samples and 81.3% among HR-HPV types), in agreement with the previous meta-analysis involving non-HIV patients^27,28^, while other HR-HPV types were also detected (HPV-18, -33, -56, and -58). These recent meta-analyses provide evidence that HPV infection may increase the risk of developing lung cancer in HIV-negative individuals, but the causal contribution of HPV infection to lung cancer is still under discussion^27,28^. Moreover, the considerable variability between studies on the prevalence of HPV DNA observed in lung cancers has led to the recommendation that care must be exercised with regard to laboratory detection methods for accurate detection of HPV infection in order to elucidate its role in lung carcinogenesis^26^.

It is well known that the sensitivity of HPV DNA detection methods could be influenced by limitations of the amplification methods (amplicon length, gene targeted, generic *vs.* type-specific primer design), as well as by the quality of the specimens tested (low DNA yield in small biopsies, low DNA quality)^26^. The short length of amplicons obtained with the INNO-LiPA and the in-house PCR methods used might have led to the increased detection rate observed in comparison with other methods, as DNA may be partially degraded during the fixation procedure of FFPE tissues^45^ leading to false negative results by methods amplifying longer regions. Unfortunately, the information regarding amplicon length is proprietary information of Seegene for the Anyplex™ II HPV28 assay. In order to diminish the potential intrinsic limitations of each of the HPV DNA detection methods, we combined the results obtained by several commercial and in-house assays targeting different HPV regions and/or based on different amplicon lengths, which could have led to the higher prevalence observed in comparison with previous studies of lung cancer. However, the presence of HR-HPV types in lung cancer in our study was confirmed by more than one assay in most samples, adding strength to our findings. While not directly comparable to our study in lung cancer archival biopsies and cytologies, in a recent study using archival FFPE biopsies from oropharyngeal carcinomas, the Anyplex™ II HPV28 assay showed a higher HPV DNA detection rate than the LiPA assay^39^. However, only four of the 18 samples that were positive by Anyplex and negative by LiPA were p16 positive and HPV mRNA was not assessed, leading the authors to discuss that false-positive results by the first assay, which was performed the last, could not be ruled out.

It is important to stress that the detection of HPV DNA in lung cancers is not synonymous with an active infection, and this finding is not sufficient to demonstrate the causal relationship with oncogenesis. In order to establish causality, expert recommendations suggest the assessment of the presence of integrated HPV genomes or expression of HPV E6/E7 oncogenes by means of mRNA detection^26^. The transcriptional activity of HPV oncogenes in lung cancer in the non-HIV-infected population has been demonstrated previously^46,47^. Here, the E6 mRNA was detected in two out of 10 (20.0%) HPV-16 DNA positive lung cancers analyzed from HIV-infected individuals. However, as we have noted, these two patients had a background of previous neoplasms due to HPV-16: an anal canal neoplasm in one case, and a vulvar neoplasm and an inguinal lymph node metastasis in the other. Therefore, those lung cancers with HPV E6 mRNA expression were not primary lung cancers. These results are in agreement with a previous study which concluded that in the event that HPV DNA is detected in lung cancer, the possibility of metastasis of squamous neoplasia should be considered^48–50^. These two samples were p16-positive, but this marker was also detected in four other samples (three of them negative for HPV). A meta-analysis of oropharyngeal carcinomas has confirmed the high sensitivity but moderate specificity of p16 to detect a transforming HPV infection^51^. Similarly, in pulmonary squamous cell carcinoma there has been shown to be no association between the presence of HPV DNA and the expression of p16 protein^43^. It has been suggested that in the absence of HPV16, p16 expression is likely due to non-HPV-related genetic or epigenetic loss of pRb.

Other factors could have been relevant in predisposing the study participants to lung cancer. Most of them were men with a history of smoking and parenteral use of drugs. Lung cancer is one of the most frequent neoplasms in HIV-positive individuals; as many of them have a long-term history of smoking or are ex-smokers, smoking may be considered the main predisposing cause. Additionally, other cofactors may have been chronic immunosuppression secondary to HIV infection, chronic pulmonary inflammation, a history of bacterial pneumonias, or emphysema^52^.

Our study has some limitations to be considered. The definition used for HPV prevalence (HPV DNA detectable by at least with one laboratory method) could have led to an overestimation. Given the limited availability of tissue sample in certain cases, not all tests were performed in all lung cancer samples, and HPV typing was not possible in six samples. Another limitation is the absence of data on the HPV negative samples, which were not analysed due to the unavailability of tissue sample. In fact, this drawback was anticipated when the study protocol was planned. Hence, the fact that this was a retrospective study based on available tissue samples previously used for lung cancer diagnoses may limit the study conclusion. The cohort was relatively small, did not include non-cancer HIV-positive controls, and only involved a single centre, which may limit the generalizability of the results beyond the population and conditions studied. However, our hospital is a referral centre for HIV clinical therapy and admits a large proportion of all local HIV patients with comorbidities.

In conclusion, a relatively high prevalence of HR-HPV DNA was found in lung cancers in the HIV-infected patients included. However, viral oncogene expression was not detected in most cases. Our results may call into question the hypothesis that HPV is involved in lung cancer oncogenesis. However, they suggest that in an HIV-infected patient with a background of a cancer related to HPV, if HR-HPV DNA is detected in the lung cancer, it should be regarded as a metastasis of the previous cancer, just as would be the case among HIV-negative individuals.

## Data Availability

The datasets generated during and/or analysed during the current study are available from the corresponding author on reasonable request. Elisa Martró (emartro@igtp.cat) will share the datasets with permission from Guillem Sirera (gsirera@flsida.org), head of HPV-HIV coinfection research team.
